# Treatment initiation among tuberculosis patients: the role of short message service (SMS) technology and Ward-based outreach teams (WBOTs)

**DOI:** 10.1186/s12889-022-12736-6

**Published:** 2022-02-15

**Authors:** Judith R. M. Mwansa-Kambafwile, Charles Chasela, Jonathan Levin, Nazir Ismail, Colin Menezes

**Affiliations:** 1 Department of Epidemiology and Biostatistics, School of Public Health, Faculty of Health Sciences, University of Witwatersrand, Johannesburg, South Africa; 2 Centre for Tuberculosis, National Institute of Communicable Diseases, Johannesburg, South Africa; 3 Fellow of the Consortium for Advanced Research Training in Africa (CARTA), Johannesburg, South Africa; 4 Division of Infectious Diseases, Department of Internal Medicine, School of Clinical Medicine, Faculty of Health Sciences, University of Witwatersrand, Johannesburg, South Africa

## Abstract

**Background:**

In South Africa, tuberculosis (TB) is a public health problem with treatment initiation failure rates varying between 14.9 and 25%. Lack of proper provider/patient communication on next steps after testing, not being aware that results are ready; and other competing priorities are some of the reasons for this failure. We aimed to assess the effectiveness of Short Message Service (SMS) technology and ward-based outreach teams (WBOTs) in improving TB treatment initiation. A 3-arm randomized controlled trial (Standard of care-SOC, SMS technology or WBOTs) was conducted between September 2018 and April 2020. Newly diagnosed TB patients randomly allocated to SMS and WBOTs groups were sent reminder messages (text message or paper slip respectively) that results were ready. Due to unforeseen challenges (financial and impact of the COVID 19 pandemic), implementation was only in two of the eight clinics planned.

**Results:**

314 TB patients were assigned to one of three groups (SOC = 104, WBOTs = 105, and SMS = 105). Chi-square tests were used to compare proportions starting treatment (primary outcome). More patients in the SMS group (92/105; 88%) initiated treatment than in the SOC group (81/104; 78%), although this difference did not reach statistical significance (*P* = 0.062). The time to treatment initiation was significantly shorter in the SMS group than in the SOC group (*P* < 0.001). The proportions of patients initiated on treatment in the WBOTs group (45/62; 73%) and in the SOC group (44/61; 72%) were similar (*P* = 0.956). The times to treatment initiation for these two groups were also similar. The 3 group analysis yielded similar proportions initiated on treatment (*P* = 0.048 for SMS/SOC comparison and P = 0.956 for WBOTs/SOC comparison) but analysis of times to treatment initiation yielded some variations.

**Conclusion:**

Reminder SMS messages sent to newly diagnosed TB patients improved the time to treatment initiation. Further research is required to show effect of the WBOTs intervention.

**Trial registration:**

Retrospectively registered with the Pan African Clinical Trial Registry (PACTR202101914895981).

The trial was registered with the Pan African Clinical Trial Registry on 25 January, 2021 (ref: PACTR202101914895981; https://pactr.samrc.ac.za). The registration was retrospective due to an oversight. Nevertheless, the protocol details outlined in our ethics application were strictly adhered to.

**Supplementary Information:**

The online version contains supplementary material available at 10.1186/s12889-022-12736-6.

## Introduction

Tuberculosis (TB) is a public health problem in South Africa. The incidence of this preventable infection is currently 615/100000 population and the HIV co-infection rate is 58% [1] . Despite a decline in mortality, TB topped the country’s list of the ten leading underlying natural causes of death in 2015–2017 [2]. Patients whose TB test results are positive but never get initiated on treatment are known as initial loss to follow up (LTFU) patients. In South Africa, initial LTFU rates range between 12 and 17.9% [3–6] but can be as high as 22% in densely populated cities with migrant populations such as the inner-city Johannesburg [7] and is even higher in rural settings [8].

South Africa’s TB targets for treatment success rate and for initial LTFU rate are greater than 90% and less than 5% respectively [9]. The former is the proportion of new positive TB patients cured, plus the number completed treatment but not meeting the criteria for “cure” or “failure”. The denominator for this is the total number of new positive pulmonary TB patients registered [9]. However, the number of patients who initiate treatment is not all those who are eligible but only a proportion of them [8, 10]. Therefore, it is possible that the success rate is an overestimate due to the assumption that the initial LTFU rate is negligible and not factored in.

Over the past decade, there has been an increase in the usage of mobile cellular phones globally with over 7 billion mobile cellular subscriptions by 2015 [11]. Reminding patients through short message service (SMS) technology has been shown to improve adherence to various chronic medications [12–16]. This technology is also acceptable among patients who use it [17, 18]. It has been shown to improve return for appointments [19, 20] as well as treatment initiation where messages with results were sent to healthcare workers [21, 22].

The model of taking healthcare services to the communities has resulted in decreased infant mortality [23] and improved access to healthcare [24]. Ward-Based Outreach Teams (WBOTs) are a cadre of staff (mostly community healthcare workers) in South Africa’s re-engineered primary healthcare (PHC) model, who offer services at household and community levels. For TB services, the teams identify, support and follow-up already diagnosed TB patients and their contacts with a minor or no role in treatment initiation [25].

We aimed to evaluate the effectiveness of the SMS technology and WBOTs in increasing the proportion who initiated treatment and in reducing the time to treatment initiation among TB patients.

## Methods

According to the South African National Tuberculosis Control Programme guidelines, presumptive TB patients who have a productive cough should produce sputum on the spot, and this specimen is sent to the laboratory for testing using the Xpert MTB/Rif (Xpert) machine if available. Alternatively, smear microscopy is used as the diagnostic test. Although the laboratory turnaround time for Xpert is theoretically 2 h, the patient is asked to come back for results after 2 days. This is to cater for the transportation time and delivery of results to the PHC facility. If the TB test result is positive and the patient comes after the 2 days, treatment is initiated the same day or within 5 days. A second sputum sample is collected in cases where the diagnosis was made using Xpert. This sample, which is sent for smear microscopy, is a baseline for monitoring before changing from intensive phase to continuation phase of treatment and at the time of discharge from treatment [9].

### Design

A randomized controlled trial (RCT) was conducted at two public sector primary level clinics in inner-city Johannesburg, South Africa. Participants were enrolled between 10 September 2018 to 25 March 2020 and the last date for follow up was 22 April 2020. The inner-city Johannesburg area has a population of 4.4 million and occupies a 1645km^2^ area [26]. Using data from the 2017 National TB Control Program (NTCP) report, the 8 clinics with the highest TB notification in the area were selected. Due to unforeseen financial challenges and the effects of the COVID 19 pandemic, the study was only implemented in two of these 8 clinics. These non-fee paying clinics service a generally low income population and offer holistic primary level healthcare services such as family planning, antenatal care and baby care among others.

### Participants

The standard clinic practice as per National TB Control Program for South Africa [9] is that all patients accessing any healthcare services in any part of the clinic are screened for TB symptoms. Those found with TB symptoms (presumptive TB patients) are sent to the TB room for testing. During the study period, such patients who were eligible and consented to participation, were enrolled. Patients aged 18 years old and above, not yet diagnosed with TB, and who had submitted sputum to the TB nurse were eligible.

### Sample size

It was assumed that the treatment initiation in this study would increase from the 82% average upper limit reported in South African studies [3–5, 7] to 95% in each of the groups with an intervention (with either SMS technology or with WBOTs) as per target of the country’s National TB Control Program [9]. Based on a power of 80% and a level of significance of 0.05 to detect an increase in treatment initiation of 13% in either of the intervention groups, we estimated a required minimum sample size of 104 positive TB patients in each group. The trial was powered to detect a difference between the SMS group and the SOC group and also to detect a difference between the WBOTs group and the SOC group, but was not powered to detect a difference between the SMS group and the WBOTs group.

### Study procedures

After obtaining written informed consent, patients meeting the inclusion criteria were interviewed using a pre-piloted structured questionnaire to obtain sociodemographic and clinical data. Their contact details, including mobile phone numbers they could be contacted on, were also recorded both in the TB case identification register and on the study data abstraction form.

To determine the level of knowledge of participants about TB, the following four questions were asked:

- “Before testing this time, had you heard about TB?”
- “Before testing this time, did you know how one can get TB?”
- “Before testing this time, did you know symptoms of TB?”
- “Before testing this time, did you know TB be can be cured?”

Knowledge level evaluation was made based on the number of “Yes” (correct) answers each patient scored. If a patient scored two or fewer correct answers, he or she was classified into the “none or little knowledge” category; and then those that scored three or four correct answers were classified in the “adequate knowledge” category.

Patients were asked to return for results after 2 days as per national guidelines [9]. Using a Stata generated pre-run block randomization sequence (block sizes ranging between 6 and 15) [27], participants who tested positive for TB were assigned to one of the three groups by a researcher not involved in participant enrolment (standard of care (SOC), WBOTs, or SMS). Patients allocated to either the SMS or the WBOTs group received reminder messages telling them that their results were ready at the facility while those in the SOC group did not receive any messages.

The reminder messages were sent through SMS messaging for those in the SMS group and through paper slip messages delivered by WBOTs for those allocated to the WBOTs group. The WBOTs were given the paper slips by the research assistants and they carried during their routine household visits. The paper slips had the physical addresses of the patients and this guided the WBOTs in locating the patients. Up to 3 attempts were made in trying to locate patients before finally labelling the patients as “not found”. Any undelivered paper slips were handed back to the study staff and were recorded. Delivery reports for the messages sent via SMS were noted.

To maintain confidentiality, the reminder message did not state details of why the person was needed at the clinic. In addition, the paper slips were delivered in sealed envelopes. The message was in English or isiZulu depending on language preference selected at study enrolment. The content was the same for both intervention groups and read as follows:

“*Good day, your results are ready at the clinic for your collection. You are advised to collect your results as soon as possible”.*

The TB case identification registers at the clinics were checked regularly for the results of the patients enrolled in the study. The names of those with a positive result were checked for in the hard copy TB treatment initiation registers where patients initiated on TB treatment are captured. This was also confirmed by checking patient details in the TB module of TIER.net electronic register as well as using hard copy TB files which are opened once a patient is initiated on treatment. Participants with positive TB test results but not initiated on treatment within 4 weeks from the date of TB test were noted. The time to treatment initiation was measured and ascertained by calculating the number of days between date of sputum submission for TB testing and the date of TB treatment initiation.

### Investigator and participant blinding

Research assistants checking for the outcomes of the patients were blinded to the different groups to which patients were allocated. They were not involved in randomisation, intervention delivery and did not have access to participant data with respective intervention groups. This minimized observer ascertainment bias. Participants were told that they would receive one of the two interventions or neither (standard of care). However, depending on the type of reminder message they received or if they did not receive any message at all, they would be aware of their allocation group. Therefore, blinding was not possible and as such, participant ascertainment bias could not be avoided.

### Study outcomes

We defined treatment initiation among TB patients as being started on TB treatment when one was diagnosed with TB. The primary outcome was the proportion initiated on treatment within 28 days from the date of submitting sputum. The secondary outcome was the time (in days) to treatment initiation.

### Statistical analysis

Data were analysed using STATA® version 14.2 software [27]. The study was not powered to compare the interventions against each other, but to compare each intervention against the standard of care [28].

To evaluate the primary outcome, we used descriptive frequency tables to determine the proportions of treatment initiation across the study groups. The proportion initiated on treatment within 28 days was estimated for each study group, and comparisons between proportions were carried out using a chi-square test. In order to estimate the ‘risk’ ratio for treatment initiation for an intervention group relative to the SOC group; and adjusting for other variables, Poisson regression models were fitted with robust estimation of standard errors, as recommended by Cummings [29]. The use of robust standard errors is suggested since the outcome (treatment initiation) is not rare, so the Poisson approximation to the binomial distribution will not be very accurate, and the usual Poisson standard errors will be too large (since essentially the Poisson distribution allows a participant to initiate TB treatment on more than one occasion). Candidate variables for the models to be used in addition to treatment group (with SOC as the reference level) were marital status, body mass index, alcohol consumption, smoking, monthly income, prior clinic consultation, comorbidities, age, gender, employment status, TB test disclosure, history of TB contact, travel time to the clinic, HIV status and severity of TB symptoms. These candidate variables were chosen from existing literature on variables known to be related to TB treatment initiation. The selection of variables in the final model was based on unadjusted analysis and forward selection of variables chosen by the unadjusted analysis.

We used Kaplan–Meier curves and the log-rank test to analyse the time to treatment initiation across the study groups. The time of submitting sputum for TB testing (date of signing informed consent) and the date of treatment initiation were used to calculate the duration of time in the study. Patients who were transferred out or died before treatment initiation were censored from the analysis. The transferred out patients were censored out at the date of transfer out, while for patients who died (and for whom the exact date of death was not known) or who were lost to follow-up, the censoring date was taken as day 29. The first reason for choosing day 29 for censoring was that research assistants checked for initiation of treatment in both the hard copy TB treatment initiation register and in the TB module of the TIER.net electronic register for 28 days following enrolment. The second reason was so that the Kaplan Meier plots will correctly show that the overall proportion of participants initiating treatment was less than 100%. We used Cox regression analysis to determine associations between treatment group and initiating treatment, adjusting for explanatory variables associated with time to initiation of treatment. The variables selected for inclusion in these models were the same variables that were used in the multivariable analysis for the primary outcome. The proportional hazards assumption was checked using the Schoenfeld residuals. A sensitivity analysis was carried out fitting a Cox regression model with censoring at day 3 rather than day 29 for those participants for whom the date of loss to follow-up was unknown. The “day 3” selection was based on the fact that the national guidelines advise that patients return for results within 2 days of TB testing [9].

## Results

Of the 3147 patients who sought TB services during the study period, 2880 were eligible for enrolment into the study, and 2850 consented to participation (99% response rate).

Eighty six percent (2448/2850) were negative while 3% (88/2850) of the results were categorized as “other result”. “Other result” category consisted of Xpert trace results as well as failed tests due to leakage or contamination.

Of the 88 patients with “other” test results, there were 41 patients with Xpert trace results. At the time of the data collection, the South African national guidelines [9] were not clear on the management of presumptive TB patients with Xpert trace results. As a result, the practice across facilities was not standardized with some facilities treating such patients as TB positive and initiating them on treatment while other facilities waited for TB culture result before confirming the diagnosis. Therefore, patients with such results were not randomized to any group.

Eleven percent (314/2850) of the participants tested positive for TB. These were randomly assigned to one of the 3 groups (SOC = 104, WBOTs = 105, and SMS = 105).

**Fig. 1:**
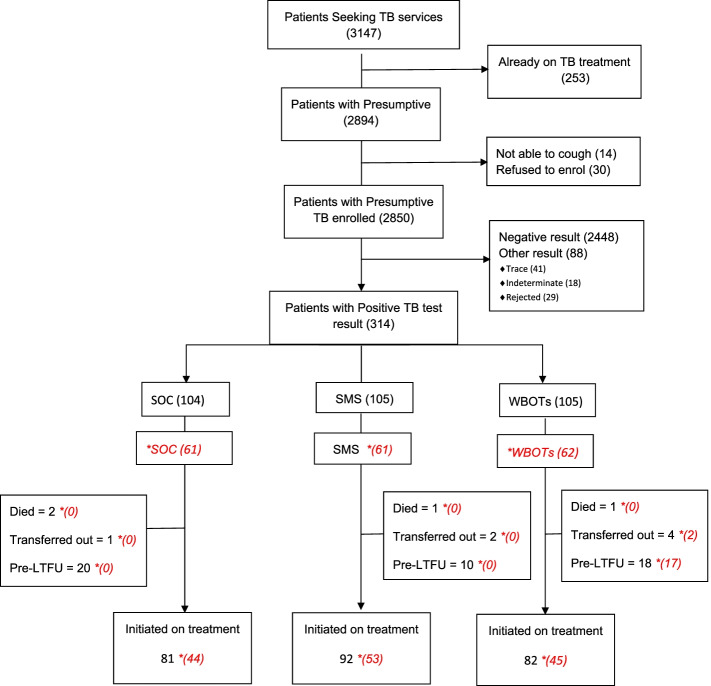
Participant Enrolment Flow Chart. *Numbers in line with implementation of WBOTs intervention

Following operational challenges because of the COVID 19 pandemic, delivery of paper slips to the participants randomized to the WBOTs group was discontinued. However, randomization of the participants to the three groups using the predetermined block randomization sequence continued. Figure 1 below illustrates the enrolment flow. The writing in *italic* is based on actual implementation numbers based on the WBOTs intervention.

### Sociodemographic characteristics

The three groups were similar in terms of sociodemographic data and therefore comparable (Table 1).

**Table 1:** Participant characteristics

Sociodemographic Characteristics	SOC (*n* = 104)	SMS (*n* = 105)	WBOTs (*n* = 105)
*Age category*			
<= 30 years n (%)	21 (20)	21 (20)	26 (25)
31 to 45 years n (%)	55 (53)	57 (54)	60 (57)
46 to 60 years n (%)	18 (17)	21 (20)	13 (12)
More than 60 years n (%)	10 (10)	6 (6)	6 (6)
Median age; years (IQR)	37 (31–49)	39 (32–46)	37 (31–42)
*BMI range*			
BMI < 18.5 n (%)	21 (20)	19 (18)	21 (20)
BMI > =18.5 & BMI < 25 n (%)	47 (45)	58 (55)	52 (49)
BMI > =25 & BMI < 30 n (%)	28 (27)	19 (18)	27 (26)
BMI > =30 n (%)	8 (8)	9 (9)	5 (5)
Median BMI; kg/m^2^ (IQR)	22.6 (19.1–26.2)	22.4 (19.4–25.1)	22.4 (19.1–26.0)
*Gender*			
Male n (%)	57 (55)	62 (59)	64 (61)
Female n (%)	47 (45)	43 (41)	41 (39)
*Marital status*			
Not married n (%)	86 (83)	87 (83)	86 (82)
Married n (%)	18 (17)	18 (17)	19 (18)
*Highest level of education attained*			
Primary or lower n (%)	29 (28)	23 (22)	25 (24)
Secondary or higher n (%)	75 (72)	82 (78)	80 (76)
*Employment*			
Not employed n (%)	45 (43)	50 (48)	47 (45)
Employed n (%)	59 (57)	55 (52)	58 (55)
*Financial status*			
Median monthly income; ZAR (IQR)	4000 (3000–6500)	4300 (3000–5500)	4400 (3500–5500)
Median # supporting financially (IQR)	1 (0–2)	1 (0–3)	1 (0–2)
*Time to clinic*			
<= 30 min	72 (69)	73 (70)	70 (67)
> 30 min	32 (31)	32 (30)	35 (33)
*Alcohol consumption*			
No	59 (57)	62 (59)	60 (57)
Yes	45 (43)	43 (41)	45 (43)
*Smoking*			
No	70 (67)	58 (55)	63 (60)
Yes	34 (33)	47 (45)	42 (40)
*TB knowledge*			
None or little (<=2 correct answers)	39 (38)	36 (34)	41 (39)
Adequate (> = 3 correct answers)	65 (62)	69 (66)	64 (61)
*Cough duration*			
Less than 2 weeks	45 (43)	47 (45)	45 (43)
2 weeks or longer	59 (57)	58 (55)	60 (57)
*Prior consultation*			
No	85 (82)	79 (75)	84 (80)
Yes	19 (18)	26 (25)	21 (20)
*Severity of TB symptoms*			
Mild	82 (79)	84 (80)	85 (81)
Not mild	22 (21)	21 (20)	20 (19)
*History of TB*			
No	86 (83)	86 (82)	83 (79)
Yes	18 (17)	19 (18)	22 (21)
*HIV status*			
Positive	52 (50)	41 (39)	43 (41)
Negative	43 (41)	41 (39)	38 (36)
Unknown	9 (9)	23 (22)	24 (23)
*On ART*^b^ *(n = 136)*			
No	33 (63)	24 (59)	21 (49)
Yes	19 (37)	17 (41)	22 (51)
*Comorbidities*			
Diabetes	6 (6)	4 (4)	6 (6)
CVS^a^ problem	12 (12)	5 (5)	2 (2)
Epilepsy	2 (2)	1 (1)	0 (0)
Asthma	1 (1)	4 (4)	0 (0)
*Comorbidities including HIV*			
No	44 (42)	60 (57)	57 (54)
Yes	60 (58)	45 (43)	48 (46)

^a^*CVS* cardiovascular, ^b^*ART* Antiretroviral therapy

At least half of the participants in all the groups had been feeling unwell for more than two weeks at the time of presentation to the facility. Over three quarters of each group’s participants had not sought medical attention for the current problem prior to study enrolment.

Overall, 43% (136/314) of the participants were HIV positive. Of these, 43% (58/136) were on ART (19 in the SOC group, 17 in the SMS group, and 22 in the WBOTs group). Thirty-one percent (18/58–5 in the SOC, 5 in the SMS, and eight in the WBOT groups) of patients on ART had been on treatment for less than one month at the time they tested for TB. The proportion of patients who were not aware of their HIV status at the time of enrolment was 18% (56/314).

Over half (30/58) of the patients on ART were not sure of the names of the drugs they were taking, and about half of these patients (53% - 16/30) had only been on treatment for less than a month.

### Analysis of the SOC and SMS groups

This section highlights results of all the 209 patients randomized to SOC group (104) and SMS group (105) during the entire study period.

#### Proportions initiated on treatment

Three of the 209 patients were transferred out while another three died before treatment initiation. Of the 209 patients, 173 (83%) were initiated on treatment. There were 92/105 (88%) in the SMS group and 81/104 (78%) in the SOC group (*P* = 0.062). Patients in the SMS group were 12% more likely to initiate treatment than those in the SOC group (RR = 1.12; 95% CI: 0.99–1.28). This effect size increased in the multivariable analysis (Table 2 and Table S1).

**Table 2:** Treatment initiation in the different groups

	UNIVARIABLE FINDINGS *N* = 209)			^a^MULTIVARIABLE FINDINGS (*N* = 209)		
	**Unadjusted IRR**	**Confidence Interval**	***p*-value**	**Adjusted IRR**	**Confidence Interval**	***p*-value**
Treatment Initiation						
*Allocation group*						
SOC	Ref					
SMS	1.12	0.99–1.28	0.066	1.15	1.02–1.31	0.026
	**Unadjusted HR**	**Confidence Interval**	***p*-value**	**Adjusted HR**	**Confidence Interval**	***p*-value**
Time to Treatment Initiation (Day 29 censoring)						
*Allocation group*						
SOC	Ref					
SMS	2.77	2.03–3.77	< 0.001	3.29	2.36–4.58	< 0.001
Time to Treatment Initiation (Day 3 censoring)						
*Allocation group*						
SOC	Ref					
SMS	4.67	3.30–6.60	< 0.001	5.05	3.48–7.33	< 0.001

^a^Adjusted for age, gender, employment status, TB test disclosure, history of TB contact, travel time to clinic, HIV status and severity of TB symptoms

#### Time to treatment initiation

At any particular time, patients in the SMS group were 2.8 times more likely to initiate treatment early than those in the SOC group (HR = 2.77; 95% CI: 2.03–3.77). This effect size increased slightly when adjusted for age, gender, employment status, TB test disclosure, history of TB contact, travel time to clinic, HIV status and severity of TB symptoms (HR = 3.29; 95% CI: 2.36–4.58) (Table 2 and Table S2). The estimates were increased when censoring was done at day 3.

**Fig. 2:**
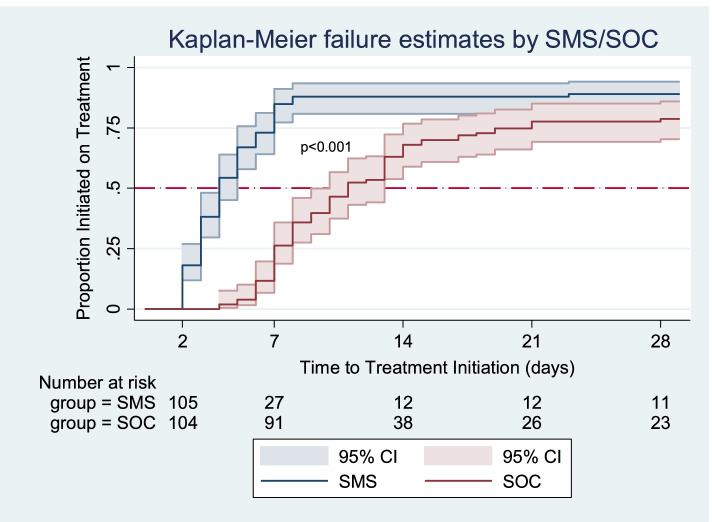
Time to treatment initiation for the SMS and SOC groups

The total analysis time at risk and under observation of the patients was 1357 days. Patients in the SMS group had a shorter time to treatment initiation than those receiving standard of care (SMS-4 days, IQR: 3–5 versus SOC- 8 days, IQR: 5–13). This difference was significant (*P* < 0.001). At least half of the patients who initiated treatment in the SMS group had done so by the 4th day while it took 8 days for those in the SOC group (Fig. 2).

### Analysis of SOC and WBOTs groups

This section highlights results for SOC and WBOTs groups but restricted to the duration when paper slip reminders were implemented. The total number of patients analysed was 123 (SOC = 61 and WBOTs = 62).

#### Proportions initiated on treatment

Three of the 123 patients were transferred out while two died before treatment initiation. Treatment was initiated in 72% (89/123) of them. The proportions in the SOC (44/61; 72%) and WBOTs (45/62; 73%) groups were similar (*P* = 0.956). The chances of initiating treatment among patients in the 2 groups were also similar (IRR = 1.01; 95% CI: 0.81–1.25). This effect reduced slightly in the multivariable analysis (Table 3 and Table S3).

**Table 3:** Treatment initiation in the different groups

	UNIVARIABLE FINDINGS (*N* = 123)			^a^MULTIVARIABLE FINDINGS (*N* = 123)		
	**Unadjusted IRR**	**Confidence Interval**	***p*-value**	**Adjusted IRR**	**Confidence Interval**	***p*-value**
Treatment Initiation						
*Allocation group*						
SOC	Ref					
WBOTs	1.01	0.81–1.25	0.956	0.97	0.76–1.25	0.830
	**Unadjusted HR**	**Confidence Interval**	***p*-value**	**Adjusted HR**	**Confidence Interval**	***p*-value**
Time to Treatment Initiation (Day 29 censoring)						
*Allocation group*						
SOC	Ref					
WBOTs	1.18	0.78–1.79	0.434	1.11	0.70–1.77	0.654
Time to Treatment Initiation (Day 3 censoring)						
*Allocation group*						
SOC	Ref					
WBOTs	1.59	1.04–2.43	0.033	1.64	0.98–2.73	0.059

^a^Adjusted for age, gender, employment status, TB test disclosure, history of TB contact, travel time to clinic, HIV status and severity of TB symptoms

#### Time to treatment initiation

At any particular time, patients in the WBOTs group were 18% more likely to initiate treatment than those in the SOC group (HR = 1.18; 95% 0.78–1.79). When adjusted for age, gender, employment status, TB test disclosure, history of TB contact, travel time to clinic, HIV status and severity of TB symptoms, the effect size was similar (HR = 1.11; 95% CI: 0.70–1.77) (Table 3 and Table S4). The estimates were increased when censoring was done at day 3.

**Fig. 3:**
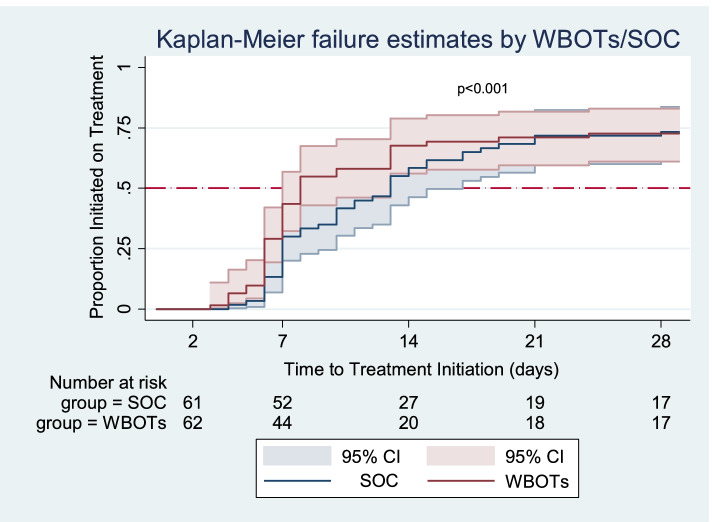
Time to treatment initiation for the SOC and WBOTs groups

The total analysis time at risk and under observation of the patients was 1809 days. Patients in the WBOTs group had a shorter time to treatment initiation (8 days, IQR: 6–29) than those in the SOC group (13 days, IQR: 7–29). This difference was significant (log-rank *P* < 0.001). At least half of the patients who initiated treatment in the WBOTs group had done so by the 8th day while it took 13 days for those in the SOC group (Fig. 3).

### Analysis of SOC, SMS and WBOTs groups

This section highlights results from all three randomization groups (SOC, SMS and WBOTs) but restricted to the duration when paper slip reminders were implemented. The total number of patients analysed was 184 (SOC = 61, SMS = 61 and WBOTs = 62).

#### Proportions initiated on treatment

Five of the 184 patients were transferred out while three died before treatment initiation. Treatment was initiated in 77% (142/184) of them. There were 53/61 (87%) in the SMS group and 44/61 (72%) in the SOC group and 45/62 (73%) in the WBOTs group (*P* = 0.087).

Patients in the SMS group were 20% more likely to initiate treatment than those in the SOC group (IRR = 1.20; 95% CI: 1.00–1.45) while those in the WBOTs group were 1% more likely to initiate treatment than those in the SOC group (IRR = 1.01; 95% CI: 0.81–1.58). However, these findings were not significant and the effect sizes for both SMS and WBOTs interventions did not differ much in the adjusted analyses (Table 4 and Table S5).

**Table 4:** Treatment initiation in the different groups

	UNIVARIABLE FINDINGS (*N* = 184)			^a^MULTIVARIABLE FINDINGS (*N* = 184)		
	**Unadjusted IRR**	**Confidence Interval**	***p*-value**	**Adjusted IRR**	**Confidence Interval**	***p*-value**
Treatment Initiation						
*Allocation group*						
SOC	Ref					
SMS	1.20	1.00–1.45	0.048	1.21	1.00–1.47	0.049
WBOTs	1.01	0.81–1.25	0.956	0.98	0.78–1.24	0.883
	**Unadjusted HR**	**Confidence Interval**	***p*-value**	**Adjusted HR**	**Confidence Interval**	***p*-value**
Time to Treatment Initiation (Day 29 censoring)						
*Allocation group*						
SOC	Ref					
SMS	3.27	2.17–4.93	< 0.001	3.53	2.27–5.48	< 0.001
WBOTs	1.14	0.75–1.73	0.531	1.11	0.71–1.72	0.657
Time to Treatment Initiation (Day 3 censoring)						
*Allocation group*						
SOC	Ref					
SMS	4.61	2.99–7.10	< 0.001	4.71	2.98–7.45	< 0.001
WBOTs	1.47	0.96–2.23	0.076	1.40	0.88–2.23	0.151

^a^Adjusted for age, gender, employment status, TB test disclosure, history of TB contact, travel time to clinic, HIV status and severity of TB symptoms

#### Time to treatment initiation

At any particular time, patients in the SMS group were 3.3 times more likely to initiate treatment earlier than those in the SOC group (HR = 3.27; 95% CI: 2.17–4.93). Patients in the WBOTs group were 14% more likely to initiate treatment than those in the SOC group. However, this finding was not significant (HR = 1.14; 95% CI: 0.75–1.73). When adjusted for age, gender, employment status, TB test disclosure, history of TB contact, travel time to clinic, HIV status and severity of TB symptoms, the effect size for the SMS group increased slightly (HR = 3.53; 95% CI: 2.27–5.48) whilst that for WBOTs decreased (HR = 1.11; 95% CI: 0.71–1.72). The estimates were increased when censoring was done at day 3. Table 4 and Table S6 show the findings in the three group comparison.

**Fig. 4:**
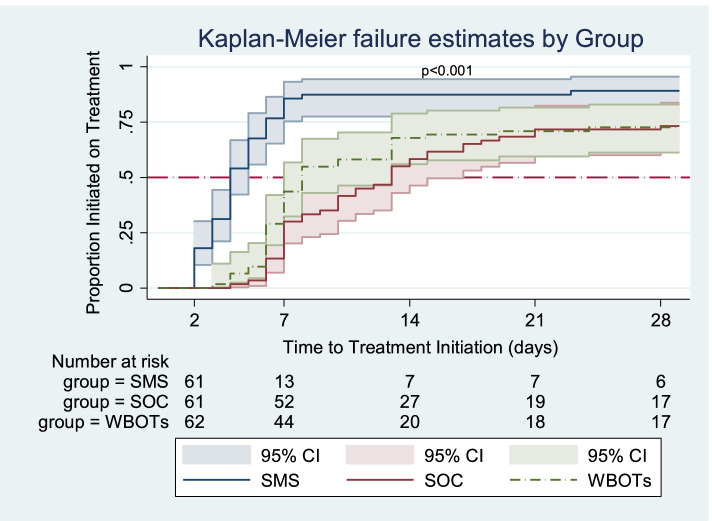
Time to treatment initiation for the SMS, SOC and WBOTs groups

The total analysis time at risk and under observation of the patients was 2242 days. Patients in the SMS group had a shorter time to treatment initiation than those receiving standard of care (SMS-4 days, IQR: 3–6 versus SOC - 13 days, IQR: 7–29). Patients in the WBOTs group also had a shorter time to treatment initiation compared to those in the SOC group (WBOTs – 8 days, IQR: 6–29 versus SOC - 13 days, IQR: 7–29). At least half of the patients who initiated treatment in the SMS group had done so by the 4th day while it took 8 and 13 days for those in the WBOTs and SOC groups respectively (Fig. 4).

## Discussion

We have some evidence that sending reminder messages to presumptive TB patients does ensure that patients diagnosed with TB are initiated on treatment. In the analysis of SMS group versus the SOC group, we found that the proportions of TB patients initiated on treatment in the two groups were similar. However, we found that the duration between sputum submission and treatment initiation across the three groups was shortest in the SMS group. Among the ones who initiated treatment in the groups, at least half of those in the SMS group had done so by the fourth day while it took 8 and 13 days for half of the participants in the WBOTs and SOC groups respectively to initiate treatment.

We found TB/HIV co-infection and ART coverage rates lower than what was reported for South Africa in the 2020 WHO Global TB Report (43% versus 58% TB/HIV co-infection rate and 43% versus 85% ART coverage rate) [1]. The low co-infection rate could be explained by the fact that a substantial proportion of patients (18% - 55/314) were not aware of their HIV status. If we suppose that at least half of these patients were actually positive, the total proportion of HIV positive patients would be similar to the national rate reported. The low ART coverage was possibly because some patients had recently been diagnosed with HIV (29% had been on ART for less than a month) and were being screened for TB before ART could be initiated.

### SMS technology

Although there has been a tremendous increase in mobile cell phone usage over the past decade [11], we were cognisant of the fact that not everyone possessed a smartphone with messaging applications other than SMS such as WhatsApp, Hangout, etc. Therefore, we used the simple SMS messaging platform to cater for people who did not have smartphones. In addition, we opted to keep the process plain and simple by sending a notification message and patients would receive the results at the facilities. Sending actual results to patients and to keep results confidential, would have added some complexity (such as using pin numbers) to the study design process. This could potentially result in patients failing to access their results as was the case in the study by Maraba and colleagues where the majority of the 20% of patients who failed to receive their results reported a lack of understanding of the process [22].

Our findings on the effectiveness of SMS technology corroborate other studies [20–22]. The patients in the SMS group were more likely to initiate treatment than those in the SOC group. This was similar to what Wagstaff and colleagues found in their study where recipients of SMS messages were more likely to return to the clinic within the requested 2 days for results than the control group [20]. Although we found a reasonable proportion of patients in the SMS group initiated on treatment, the 12% loss to follow up before treatment initiation was still higher than the 5% national target [9]. Therefore, there is need for more effort (both on a patient level and on a healthcare facility level) to ensure that all patients diagnosed with TB are initiated on treatment appropriately.

### WBOTs paper slips

Our results show similar proportions of patients initiated on treatment (73% versus 72%) and similar durations between testing and treatment initiation (8 days versus 13 days) in the WBOTs and SOC groups respectively. It is important to consider what the work of the WBOTs entails. The scope of TB program related work of the WBOTs puts emphasis on TB screening during household visits for possible sputum testing referral to respective facilities; and on treatment adherence for those already initiated on treatment [25, 30]. There is no explicit documentation in their scope of work that speaks to their role in ensuring treatment initiation among all patients diagnosed with TB. Their role is limited to referring symptomatic patients they find during home visits for testing and to ensuring treatment adherence among those already on treatment [25, 30]. A study conducted among WBOTs and TB program managers revealed that integration of the two programs through regular meetings could improve treatment initiation among TB patients [30].

WBOTs have the potential to play a key role in ensuring treatment initiation among TB patients in communities. In some settings, this cadre of healthcare staff has been pivotal in taking healthcare services to the community level and has contributed tremendously to reduction in infant mortality as well as to general good health status of the population through improved access to healthcare services [23, 24].

Although we relied on and utilized the schedule of the WBOTs to send out the paper slip reminders, we tried to emphasize the importance of the study. We also conducted a revision session on the basics of TB since gaps in TB knowledge among community healthcare workers have been found [31, 32] and scores in TB clinical knowledge and skills do worsen with an increase in the time since last training [33]. A South African study showed that community healthcare workers are willing to conduct TB related work but they do require ongoing tailor-made training and access to TB information materials [34]. With adequate capacity building, empowerment and support, the WBOTs can hugely contribute to the success of the TB program.

The SMS and WBOTs interventions are applicable to settings similar to ours. They are also relevant and may be applicable to other settings. They focus on addressing some of the patient-related and healthcare system-related reasons for failure to initiate TB treatment such as lack of communication and forgetfulness.

## Conclusion

Reminder messages to patients do play an important role in TB treatment initiation. SMS messaging is an affordable, feasible option that national TB programs can use. There is need for further research to show effect of WBOTs since implementation of this intervention was suboptimal (fewer patients than planned were exposed to the intervention in this trial). With proper integration of TB and WBOTs programs, WBOTs have the potential to contribute to improved treatment initiation.

### Limitations

The WBOTs’ work schedule was not managed by the study but rather that the teams followed their daily routine schedule. This meant that despite efforts to emphasize the importance of the study, implementation of the paper slip reminder messages was compromised. In addition, it was during the last 6 months of the data collection period that the COVID 19 pandemic started and this further made it impossible to use the WBOTs to deliver the paper slips.

Another limitation in the study was that there was no way of ascertaining if the messages reached the targeted people. It is also possible that some patients might not have gone to collect their test results at the facility where the TB test was done but went elsewhere. If positive, there was no way of knowing whether they initiated treatment especially if they changed cell phone numbers where they could be reached to ascertain this. Checking treatment registers in the facilities within inner-city Johannesburg as well as the ETR.net/TIER.net was not as easy we had anticipated due to the lack of real-time data capturing into these electronic databases. Some patients might have left the region making tracing even more impossible practically.

Although the sample size was not large and each TB patient was individually checked for in the data sources available, it is possible to have missed some TB positive participants and thereby potentially under-ascertaining treatment initiation. Re-interviewing a sample of participants would have guaranteed optimal quality assurance of the data linkage.

We recognize that sometimes phone sharing can have an impact whether or not the patient sees the message in that the person opening the message may not be the intended recipient and they may forget to convey the message.

We are unable to ascertain the impact of our interventions on treatment completion as we did not follow up with patients for the duration of their respective treatment courses.

## Supplementary Information

**Additional file 1: Table S1**: Treatment initiation in SMS and SOC groups. **Table S2**: Time to treatment initiation in SMS and SOC groups. **Table S3**: Treatment initiation in WBOTs and SOC groups. **Table S4**: Time to treatment initiation in WBOTs and SOC groups. **Table S5**: Treatment initiation in SMS, WBOTs and SOC groups. **Table S6**: Time to treatment initiation in SMS, WBOTs and SOC groups.

## Data Availability

The data has patient identifying information and therefore is not publicly available due to confidentiality agreements with the participants. However, contact with corresponding author can be made for data access.
