# Correction to: Treatment initiation among tuberculosis patients: the role of short message service (SMS) technology and Ward-based outreach teams (WBOTs)

**DOI:** 10.1186/s12889-022-12934-2

**Published:** 2022-03-18

**Authors:** Judith R. M. Mwansa-Kambafwile, Charles Chasela, Jonathan Levin, Nazir Ismail, Colin Menezes

**Affiliations:** 1 Department of Epidemiology and Biostatistics, School of Public Health, Faculty of Health Sciences, University of Witwatersrand, Johannesburg, South Africa; 2 Centre for Tuberculosis, National Institute of Communicable Diseases, Johannesburg, South Africa; 3 Fellow of the Consortium for Advanced Research Training in Africa (CARTA), Johannesburg, South Africa; 4 Division of Infectious Diseases, Department of Internal Medicine, School of Clinical Medicine, Faculty of Health Sciences, University of Witwatersrand, Johannesburg, South Africa

**Correction to: BMC Public Health 22, 318 (2022)**

**https://doi.org/10.1186/s12889-022-12736-6**

In the original publication there was an incorrect funding acknowledgement. In this correction article the correct and incorrect funding acknowledgement are published. The original article has been updated.

**Incorrect funding**

- This research was supported by the Consortium for Advanced Research Training in Africa (CARTA). CARTA is jointly led by the African Population and Health Research Center and the University of the Witwatersrand and funded by the Carnegie Corporation of New York (Grant No--B 8606.R02), Sida (Grant No:54100113), the DELTAS Africa Initiative (Grant No: 107768/Z/15/Z) and Deutscher Akademischer Austauschdienst (DAAD). The DELTAS Africa Initiative is an independent funding scheme of the African Academy of Sciences (AAS)‘s Alliance for Accelerating Excellence in Science in Africa (AESA) and supported by the New Partnership for Africa’s Development Planning and Coordinating Agency (NEPAD Agency) with funding from the Wellcome Trust (UK) and the UK government. The statements made and views expressed are solely the responsibility of the Fellow.

**Correct funding**

- This research was supported by the Consortium for Advanced Research Training in Africa (CARTA). CARTA is jointly led by the African Population and Health Research Center and the University of the Witwatersrand and funded by the Carnegie Corporation of New York (Grant No—G-19-57145), Sida (Grant No:54100113), Uppsala Monitoring Centre and the DELTAS Africa Initiative (Grant No: 107768/Z/15/Z). The DELTAS Africa Initiative is an independent funding scheme of the African Academy of Sciences (AAS)’s Alliance for Accelerating Excellence in Science in Africa (AESA) and supported by the New Partnership for Africa’s Development Planning and Coordinating Agency (NEPAD Agency) with funding from the Wellcome Trust (UK) and the UK government. The statements made and views expressed are solely the responsibility of the Fellow.

