# Supplementary material for: Treatment initiation among tuberculosis patients: the role of short message service (SMS) technology and Ward-based outreach teams (WBOTs)
Source: BMC Public Health. 2022 Feb 15;22:318. doi: 10.1186/s12889-022-12736-6 (PMC8848795; doi:10.1186/s12889-022-12736-6)
Supplement: Supplementary file 1 — Additional file 1: Table S1: Treatment initiation in SMS and SOC groups. Table S2: Time to treatment initiation in SMS and SOC groups. Table S3: Treatment initiation in WBOTs and SOC groups. Table S4: Time to treatment initiation in WBOTs and SOC groups. Table S5: Treatment initiation in SMS, WBOTs and SOC groups. Table S6: Time to treatment initiation in SMS, WBOTs and SOC groups. [file 12889_2022_12736_MOESM1_ESM.docx]

**SUPPLEMENTARY TABLES**

**SMS versus SOC**

*Table S1: Proportions initiated on treatment in SMS and SOC groups*

| UNIVARIATE FINDINGS (N = 209) | | | |
| --- | --- | --- | --- |
|  | **Unadjusted IRR** | **Confidence Interval** | **p-value** |
| *Allocation group* | | | |
| SOC | Ref |  |  |
| SMS | 1.12 | 0.99 – 1.27 | 0.066 |
| MULTIVARIATE FINDINGS (N = 209) | | | |
|  | **Adjusted IRR** | **Confidence Interval** | **p-value** |
| *Allocation group* | | | |
| SOC | Ref |  |  |
| SMS | 1.15 | 1.02 – 1.31 | 0.026 |
| *Age (years)* |  |  |  |
| 18 to 30 | Ref |  |  |
| 31 to 45 | 1.00 | 0.87 – 1.15 | 0.992 |
| 46 to 60 | 0.81 | 0.64 – 1.03 | 0.089 |
| More than 60 | 1.04 | 0.83 – 1.31 | 0.712 |
| *Gender* |  |  |  |
| Female | Ref |  |  |
| Male | 0.96 | 0.85 – 1.08 | 0.469 |
| *Employment status* |  |  |  |
| Unemployed | Ref |  |  |
| Employed | 1.01 | 0.89 – 1.15 | 0.825 |
| *Clinic visit disclosure* |  |  |  |
| Did not disclose | Ref |  |  |
| Disclosed | 1.11 | 0.98 – 1.26 | 0.106 |
| *History of TB contact* |  |  |  |
| No | Ref |  |  |
| Yes | 1.11 | 0.97 – 1.28 | 0.140 |
| *Time to clinic* |  |  |  |
| <= 30 minutes | Ref |  |  |
| > 30 minutes | 1.01 | 0.89 – 1.14 | 0.880 |
| *HIV status* |  |  |  |
| Negative | Ref |  |  |
| Positive | 1.05 | 0.92 - 1.20 | 0.450 |
| Unknown | 0.95 | 0.77 – 1.19 | 0.668 |
| *Severity of TB symptoms* |  |  |  |
| Mild | Ref |  |  |
| Not mild | 1.03 | 0.90 – 1.19 | 0.638 |

*Table S2: Time to treatment initiation in SMS and SOC groups*

| UNIVARIATE FINDINGS (N = 209) | | | |
| --- | --- | --- | --- |
|  | **Unadjusted HR** | **Confidence Interval** | **p-value** |
| *Allocation group* | | | |
| SOC | Ref |  |  |
| SMS | 2.77 | 2.03 – 3.77 | <0.001 |
| MULTIVARIATE FINDINGS (N = 209) | | | |
|  | **Adjusted HR** | **Confidence Interval** | **p-value** |
| *Allocation group* | | | |
| SOC | Ref |  |  |
| SMS | 3.29 | 2.36 – 4.58 | <0.001 |
| *Age (years)* |  |  |  |
| 18 to 30 | Ref |  |  |
| 31 to 45 | 0.91 | 0.61 – 1.37 | 0.656 |
| 46 to 60 | 0.60 | 0.35 – 1.01 | 0.054 |
| More than 60 | 1.48 | 0.76 – 2.88 | 0.251 |
| *Gender* |  |  |  |
| Female | Ref |  |  |
| Male | 0.93 | 0.67 – 1.30 | 0.665 |
| *Employment status* |  |  |  |
| Unemployed | Ref |  |  |
| Employed | 1.04 | 0.74 – 1.47 | 0.804 |
| *Clinic visit disclosure* |  |  |  |
| Did not disclose | Ref |  |  |
| Disclosed | 1.36 | 0.99 – 1.87 | 0.061 |
| *History of TB contact* |  |  |  |
| No | Ref |  |  |
| Yes | 1.45 | 0.96 – 2.18 | 0.078 |
| *Time to clinic* |  |  |  |
| <= 30 minutes | Ref |  |  |
| > 30 minutes | 0.90 | 0.64 – 1.26 | 0.529 |
| *HIV status* |  |  |  |
| Negative | Ref |  |  |
| Positive | 1.10 | 0.79 - 1.53 | 0.590 |
| Unknown | 1.06 | 0.66 – 1.70 | 0.812 |
| *Severity of TB symptoms* |  |  |  |
| Mild | Ref |  |  |
| Not mild | 1.17 | 0.80 – 1.70 | 0.421 |

**WBOTs versus SOC**

*Table S3: Proportions initiated on treatment in WBOTs and SOC groups*

| UNIVARIATE FINDINGS (N = 123) | | | |
| --- | --- | --- | --- |
|  | **Unadjusted IRR** | **Confidence Interval** | **p-value** |
| *Allocation group* | | | |
| SOC | Ref |  |  |
| WBOTs | 1.03 | 0.83 – 1.28 | 0.797 |
| MULTIVARIATE FINDINGS (N = 123) | | | |
|  | **Adjusted IRR** | **Confidence Interval** | **p-value** |
| *Allocation group* | | | |
| SOC | Ref |  |  |
| WBOTs | 1.01 | 0.79 – 1.28 | 0.961 |
| *Age (years)* |  |  |  |
| 18 to 30 | Ref |  |  |
| 31 to 45 | 0.96 | 0.74 – 1.24 | 0.749 |
| 46 to 60 | 0.86 | 0.57 – 1.30 | 0.468 |
| More than 60 | 0.79 | 0.35 – 1.78 | 0.575 |
| *Gender* |  |  |  |
| Female | Ref |  |  |
| Male | 1.05 | 0.82 – 1.35 | 0.696 |
| *Employment status* |  |  |  |
| Unemployed | Ref |  |  |
| Employed | 1.12 | 0.87 – 1.43 | 0.374 |
| *Clinic visit disclosure* |  |  |  |
| Did not disclose | Ref |  |  |
| Disclosed | 1.12 | 0.89 – 1.42 | 0.324 |
| *History of TB contact* |  |  |  |
| No | Ref |  |  |
| Yes | 1.25 | 1.00 – 1.55 | 0.046 |
| *Time to clinic* |  |  |  |
| <= 30 minutes | Ref |  |  |
| > 30 minutes | 1.04 | 0.81 – 1.32 | 0.775 |
| *HIV status* |  |  |  |
| Negative | Ref |  |  |
| Positive | 1.09 | 0.84 - 1.42 | 0.528 |
| Unknown | 1.07 | 0.75 – 1.52 | 0.723 |
| *Severity of TB symptoms* |  |  |  |
| Mild | Ref |  |  |
| Not mild | 1.03 | 0.81 – 1.31 | 0.808 |

*Table S4: Time to treatment initiation in WBOTs and SOC groups*

| UNIVARIATE FINDINGS (N = 123) | | | |
| --- | --- | --- | --- |
|  | **Unadjusted HR** | **Confidence Interval** | **p-value** |
| *Allocation group* | | | |
| SOC | Ref |  |  |
| WBOTs | 1.18 | 0.78 – 1.79 | 0.434 |
| MULTIVARIATE FINDINGS (N = 123) | | | |
|  | **Adjusted HR** | **Confidence Interval** | **p-value** |
| *Allocation group* | | | |
| SOC | Ref |  |  |
| WBOTs | 1.11 | 0.70 - 1.77 | 0.654 |
| *Age (years)* |  |  |  |
| 18 to 30 | Ref |  |  |
| 31 to 45 | 0.89 | 0.53 – 1.50 | 0.666 |
| 46 to 60 | 0.66 | 0.32 – 1.38 | 0.271 |
| More than 60 | 1.24 | 0.34 – 4.51 | 0.744 |
| *Gender* |  |  |  |
| Female | Ref |  |  |
| Male | 1.01 | 0.64 – 1.61 | 0.956 |
| *Employment status* |  |  |  |
| Unemployed | Ref |  |  |
| Employed | 1.18 | 0.74 – 1.89 | 0.486 |
| *Clinic visit disclosure* |  |  |  |
| Did not disclose | Ref |  |  |
| Disclosed | 1.28 | 0.82 – 2.00 | 0.273 |
| *History of TB contact* |  |  |  |
| No | Ref |  |  |
| Yes | 1.17 | 0.68 – 2.01 | 0.566 |
| *Time to clinic* |  |  |  |
| <= 30 minutes | Ref |  |  |
| > 30 minutes | 1.10 | 0.67 – 1.82 | 0.698 |
| *HIV status* |  |  |  |
| Negative | Ref |  |  |
| Positive | 1.21 | 0.74 – 2.00 | 0.458 |
| Unknown | 1.20 | 0.62 – 2.32 | 0.597 |
| *Severity of TB symptoms* |  |  |  |
| Mild | Ref |  |  |
| Not mild | 1.06 | 0.64 – 1.77 | 0.838 |

**ALL 3 GROUPS**

*Table S5: Proportions initiated on treatment in SMS, WBOTs and SOC groups*

| UNIVARIATE FINDINGS (N = 184) | | | |
| --- | --- | --- | --- |
|  | **Unadjusted IRR** | **Confidence Interval** | **p-value** |
| *Allocation group* | | | |
| SOC | Ref |  |  |
| SMS | 1.20 | 1.00 – 1.45 | 0.048 |
| WBOTs | 1.03 | 0.83 – 1.28 | 0.797 |
| MULTIVARIATE FINDINGS (N = 184) | | | |
|  | **Adjusted IRR** | **Confidence Interval** | **p-value** |
| *Allocation group* | | | |
| SOC | Ref |  |  |
| SMS | 1.22 | 1.01 – 1.47 | 0.043 |
| WBOTs | 1.01 | 0.81 – 1.27 | 0.931 |
| *Age (years)* |  |  |  |
| 18 to 30 | Ref |  |  |
| 31 to 45 | 0.96 | 0.80 – 1.15 | 0.630 |
| 46 to 60 | 0.82 | 0.60 – 1.11 | 0.201 |
| More than 60 | 0.95 | 0.64 – 1.41 | 0.808 |
| *Gender* |  |  |  |
| Female | Ref |  |  |
| Male | 1.04 | 0.88 – 1.23 | 0.657 |
| *Employment status* |  |  |  |
| Unemployed | Ref |  |  |
| Employed | 1.11 | 0.93 – 1.32 | 0.258 |
| *Clinic visit disclosure* |  |  |  |
| Did not disclose | Ref |  |  |
| Disclosed | 1.09 | 0.92 – 1.29 | 0.306 |
| *History of TB contact* |  |  |  |
| No | Ref |  |  |
| Yes | 1.18 | 0.97 – 1.43 | 0.102 |
| *Time to clinic* |  |  |  |
| <= 30 minutes | Ref |  |  |
| > 30 minutes | 0.96 | 0.82 – 1.13 | 0.652 |
| *HIV status* |  |  |  |
| Negative | Ref |  |  |
| Positive | 1.05 | 0.88 - 1.25 | 0.626 |
| Unknown | 1.04 | 0.82 – 1.33 | 0.728 |
| *Severity of TB symptoms* |  |  |  |
| Mild | Ref |  |  |
| Not mild | 1.08 | 0.92 – 1.27 | 0.319 |

*Table S6: Time to treatment initiation in SMS, WBOTs and SOC groups*

| UNIVARIATE FINDINGS (N = 184) | | | |
| --- | --- | --- | --- |
|  | **Unadjusted HR** | **Confidence Interval** | **p-value** |
| *Allocation group* | | | |
| SOC | Ref |  |  |
| SMS | 3.27 | 2.17 – 4.93 | <0.001 |
| WBOTs | 1.14 | 0.75 – 1.73 | 0.531 |
| MULTIVARIATE FINDINGS (N = 184) | | | |
|  | **Adjusted HR** | **Confidence Interval** | **p-value** |
| *Allocation group* | | | |
| SOC | Ref |  |  |
| SMS | 3.53 | 2.27 – 5.48 | <0.001 |
| WBOTs | 1.11 | 0.71 - 1.72 | 0.657 |
| *Age (years)* |  |  |  |
| 18 to 30 | Ref |  |  |
| 31 to 45 | 0.88 | 0.58 – 1.35 | 0.567 |
| 46 to 60 | 0.65 | 0.36 – 1.17 | 0.149 |
| More than 60 | 1.69 | 0.73 – 3.90 | 0.222 |
| *Gender* |  |  |  |
| Female | Ref |  |  |
| Male | 1.04 | 0.73 – 1.48 | 0.829 |
| *Employment status* |  |  |  |
| Unemployed | Ref |  |  |
| Employed | 1.12 | 0.78 – 1.62 | 0.537 |
| *Clinic visit disclosure* |  |  |  |
| Did not disclose | Ref |  |  |
| Disclosed | 1.39 | 0.97 – 1.98 | 0.071 |
| *History of TB contact* |  |  |  |
| No | Ref |  |  |
| Yes | 1.19 | 0.75 – 1.87 | 0.458 |
| *Time to clinic* |  |  |  |
| <= 30 minutes | Ref |  |  |
| > 30 minutes | 0.86 | 0.58 – 1.29 | 0.474 |
| *HIV status* |  |  |  |
| Negative | Ref |  |  |
| Positive | 1.00 | 0.67 - 1.48 | 0.989 |
| Unknown | 1.04 | 0.62 – 1.73 | 0.896 |
| *Severity of TB symptoms* |  |  |  |
| Mild | Ref |  |  |
| Not mild | 1.18 | 0.79 – 1.77 | 0.418 |
